# Herbivory in Spiders: The Importance of Pollen for Orb-Weavers

**DOI:** 10.1371/journal.pone.0082637

**Published:** 2013-11-29

**Authors:** Benjamin Eggs, Dirk Sanders

**Affiliations:** 1 Institute of Ecology and Evolution, University of Bern, Bern, Switzerland; 2 Centre for Ecology and Conservation, University of Exeter, Penryn, United Kingdom; University of California, Berkeley, United States of America

## Abstract

Orb-weaving spiders (Araneidae) are commonly regarded as generalist insect predators but resources provided by plants such as pollen may be an important dietary supplementation. Their webs snare insect prey, but can also trap aerial plankton like pollen and fungal spores. When recycling their orb webs, the spiders may therefore also feed on adhering pollen grains or fungal spores via extraoral digestion. In this study we measured stable isotope ratios in the bodies of two araneid species (*Aculepeira ceropegia* and *Araneus diadematus*), their potential prey and pollen to determine the relative contribution of pollen to their diet. We found that about 25% of juvenile orb-weaving spiders’ diet consisted of pollen, the other 75% of flying insects, mainly small dipterans and hymenopterans. The pollen grains in our study were too large to be taken up accidentally by the spiders and had first to be digested extraorally by enzymes in an active act of consumption. Therefore, pollen can be seen as a substantial component of the spiders’ diet. This finding suggests that these spiders need to be classified as omnivores rather than pure carnivores.

## Introduction

Most spiders are generalist predators preying on a wide range of other arthropods [1,2], but there is also evidence that resources directly provided by plants, such as pollen and nectar, may be an important dietary supplement [3–6].

Orb-weaving spiders (Araneidae) take down and eat their webs at regular intervals, which enables them to recycle the silk proteins efficiently [7]. As the webs are not only a snare for arthropods but also trap aerial plankton, orb-weaving spiders may also feed on adhered spores and pollen when recycling their webs [4]. Ludy & Lang [8] counted an average of 6.9 pollen grains per cm^2^ in the webs of juvenile *Araneus diadematus* Clerck within a maize field and 2.6 pollen grains per cm^2^ web area in the field margin. Smith & Mommsen [3] reported that spiderlings in a feeding experiment lived almost twice as long when fed with pollen compared to individuals fed with fungal spores or starved individuals, and about a third longer than spiders fed with aphids. They proposed that pollen might be the main food of juvenile orb-weaving spiders, with insects providing only a dietary supplement. Similarly, del Fiol et al. [4] suggested that pollen grains are energetically important during spring when insects are rare.

If the pollen grains were small in size the actual uptake of pollen might not include an active feeding as the pollen just sticks to the spider’s silk and is more or less accidentally ingested. However, pollen varies in size, structure, nutritional value and digestibility [5], with some pollen grains being too large (20 to 50 μm in diameter) to pass through the cuticular platelets of the spider’s pharynx and having a hard exterior wall [9]. As all particles larger than 1 μm are filtered out passively [7], spiders have to solve this problem by dissolving the outer coating of a pollen grain via extraoral digestion through various digestive enzymes and, then suck up the dissolved nutrients [3,5,8]. Therefore, feeding on larger pollen grains can only be achieved by active consumption, which would then need to be regarded as herbivory, and these spiders as omnivores as they feed on insect prey and pollen.

Based on the evidence from the literature, we hypothesized, that pollen is a substantial component of the diet of juvenile orb-weaving spiders; also it remains unclear to which extent it is incorporated into the spiders’ body tissues. We therefore used a stable isotope analysis, a method to research long-term nutrition, trophic level positioning and links of food webs [10,11], to provide the first direct proof of pollen consumption and its importance in araneids under natural conditions. We first investigated whether orb-weaving spiders include components of pollen that has been dusted on their webs into their body tissue. If this leads to a change in their stable isotope signature, the analysis of stable isotopes can be used to detect the importance of pollen in the diet of spiders in the field. We performed a laboratory feeding experiment with diet analysis of juvenile *Aculepeira ceropegia* (Walckenaer) and a natural diet analysis of juvenile *A. diadematus*.

## Methods and Materials

### Feeding experiment

To test whether orb-weaving spiders (Araneidae) indeed incorporate pollen into their body tissue when insect prey is also available, we provided both pollen and insects (fruit flies) in a laboratory experiment and analysed the spiders’ body tissue in a subsequent stable isotope analysis. Spiders were kept in converted 20 cardboard boxes (30*18*10 cm) with air slots on the top and at both sides for ventilation and a transparent film covering the front. Inside each box a reed-made frame provided a structure for attaching the orb-web. A Petri dish filled with humid cotton increased the air moisture. For the experiment we collected juvenile *A. ceropegia* on the 14^th^ of April 2012 on a fallow near to Uettligen/Bern (Switzerland). No specific permissions were required for sampling and no endangered or protected species were involved in our study. The spiderlings caught had an isotopic signature (mean ± SD, based on 7 individuals) of δ^13^C: -26.40 ± 0.40‰; δ^15^N: 7.98 ± 0.81‰, which was then influenced by feeding treatments.

We separated the spiders into two groups of 10 individuals: the treatment group was fed with common fruit flies (*Drosophila melanogaster* Meigen) and birch pollen (*Betula pendula* Roth; size of a pollen grain: 25 μm) dusted onto their webs, whereas the control group was fed with fruit flies only. We provided water and fed them with two fruit flies three times a week. Whenever a spiderling of the treatment group had built a web, it was sprinkled with birch pollen (about 4-5 mg). The webs were densely coated with pollen afterwards. Most spiders recycled their web every second or third day, but some individuals weaved very irregularly and infrequently, resulting in an unequal number of pollen feeding events (from 2 to 10). The experiment lasted from 24^th^ of April 2012 until 24^th^ of May 2012. Two spiderlings died within this time (one of each group) and were therefore not considered in the analysis. We weighed the spiders before and after the experiment.

### Stable isotope analysis of field collected spiders and their potential resources

We collected juvenile spiders and all potential prey items including pollen to understand the importance of the different resources for the spiders’ diet. Sampling was done on 4 days (14^th^, 18^th^, 22^nd^ and 24^th^) in May 2012 at a site near the Institute of Ecology and Evolution with spruce trees (*Picea abies* Karst) and at a pine-lined (*Pinus sylvestris* L.) lane in Bern. The most common orb-weaving spider in these areas was *A. diadematus*. We collected all the spiders, which built their webs directly within the branchwood of the trees. We also took their webs to analyse the pollen content. All the spiders were juveniles. To sample possible prey species, especially flying insects, we used sweep netting and suction sampling (on 4 days in May for 4 times (each 2 min)). Samples were taken from the branchwood of the trees and the surrounding vegetation to cover all arthropods, which can potentially be caught in an orb web. The invertebrates were identified on family level or on suborder level in the Hymenoptera and Diptera. The spruce pollen (size of a pollen grain: 75 μm) and the pine pollen (size of a pollen grain: 50 μm) was gathered by collecting some stamens from the trees and dried at room temperature.

### Procedure of the stable isotope analysis

The stable isotope analysis provides information about trophic relationships and reflects what an organism has eaten over time [10–12], because the stable isotope ratios in consumer tissues are tightly linked to those of their diet. The ratio of heavy to light stable nitrogen isotope (^15^N/^14^N) increases in a stepwise manner with each trophic level due to preferential processing of ^15^N compounds [13], whereas the ratio of the stable carbon isotopes (^13^C/^12^C) only alters slightly with each trophic level, but reflects the primary carbon source within a food web [14]. All values are reported against international standards in delta (δ) units parts per thousand (‰) [12].

Only the prosoma of the spiderlings (total of 13 individuals) was used for the stable isotope analysis to avoid any bias due to a very recent meal in the digestive tract in the opisthosoma [12]. Several individuals of small sized insects (Aphidoidae, small Hymenoptera, Nematocera and groups of small Brachycera) were grouped into one sample for the stable isotope analysis, while for large insects we used only the thorax. We assigned the different dipterans in the samples to three trophic groups per site based on similarity in stable isotope values.

### Statistical analysis

All statistical analyses were performed in R version 2.15.2 [15]. The R-package “modeest” [14] was used for estimation of Bayesian ellipses. Resource use in the spiders was analysed with Bayesian mixing models using the package “siar” [16]. We used a Trophic Enrichment Factor (TEF) of 2‰ δ^15^N per trophic level and a fractionation of 0.5‰ δ^13^C. These TEF’s are based on laboratory feeding experiments with wolf spiders by Oelbermann & Scheu [13] and Wise et al. [17]. We performed a sensitivity analysis to test whether our results were robust to changes in TEFs, covering a range of TEFs from 0 to 1 for δ^13^C and 1 to 3 for δ^15^N without considerable changes in the outputs (pollen consumption ranged from 20-40% and 12-15% for spiders from the spruce site and the pine site respectively). The impact of treatments and pollen feeding events (treatment-group only) on the increase in spider weight (difference between begin and end of experiment for treatment and control group) was tested using generalized linear models (glm) on square-root-transformed data assuming a Gaussian distribution.

## Results

### Feeding experiment

The number of pollen feeding events did not affect the gain in weight of the *A. ceropegia* spiderlings in the treatment group (glm: t_1;7_ = 0.334, p = 0.750) and treatment and control group did not differ (glm: t_1;16_ = -0.22, p = 0.83).

**Figure 1:**
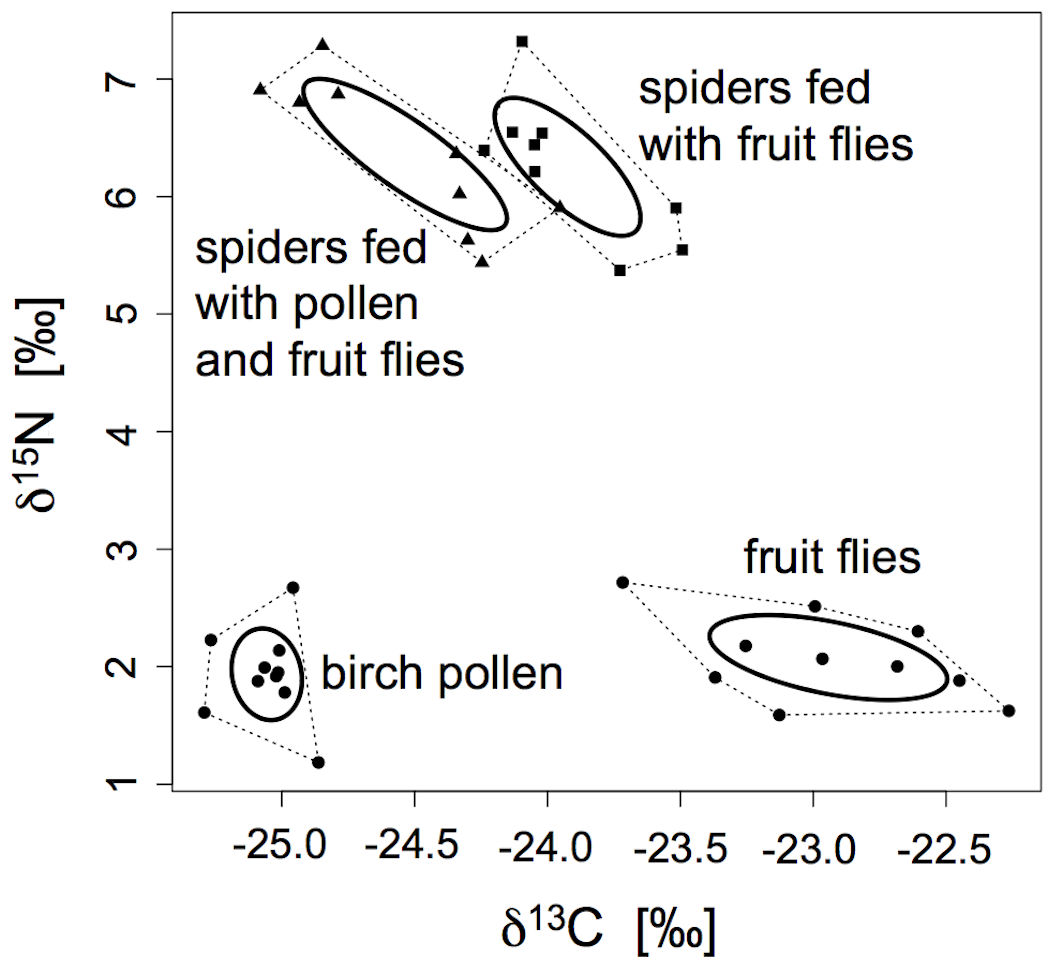
δ^15^N and δ^13^C values of juvenile *A. ceropegia* in the feeding experiment. With stable isotope Bayesian ellipses (solid line) and convex hull (dotted line) for each group. Resources (birch pollen and fruit flies) are depicted as circles, spiders fed with pollen and fruit flies as triangles and spiders only fed with fruit flies as squares.

Spiders that were fed with pollen and fruit flies had a significantly higher δ^13^C value than spiders fed with fruit flies only (treatment group: mean ± SD = -24.54 ± 0.38‰; control group: -23.92 ± 0.27‰; t_1;18_ = -3.89, p = 0.0013), whereas δ^15^N values were very similar in the two spider groups (treatment group: 6.35 ± 0.64‰; control group: 6.25 ± 0.59‰; t_1;18_ = -0.36, p = 0.724). The two stable isotope Bayesian ellipses did not overlap (Figure 1), which is clear evidence that pollen feeding leads to a change in the stable isotope signature in orb-weaving spiders. In the treatments, an average of 38.02 ± 10.22% of the spider’s diet consisted of pollen.

### Stable isotope analysis of field collected spiders and their potential resources

The study sites harboured different arthropod species (Table 1.), but the small dipterans were by far the most common arthropods in both environments. All webs were covered with pollen, most likely from the trees in which the spiders had built their webs.

**Table 1:** Stable isotope diet analysis of field collected *A. diadematus* and their potential resources.

**Habitat**	**Sample**	**n**	**δ^13^C**	**δ^15^N**
Spruce site	*Araneus diadematus*	8	-26.59 ± 0.81‰	6.06 ± 1.17‰
Spruce site	Pollen *Picea abies*	10	-26.27 ± 0.07‰	1.59 ± 0.41‰
Spruce site	Aphididae	5	-31.91 ± 0.29‰	1.43 ± 0.24‰
Spruce site	Hymenoptera	4	-26.71 ± 0.34‰	4.11 ± 3.45‰
Spruce site	Nematocera	5	-28.01 ± 0.64‰	1.68 ± 2.36‰
Spruce site	Brachycera A	5	-24.70 ± 0.54‰	14.15 ± 1.57‰
Spruce site	Brachycera B	4	-29.56 ± 0.97‰	3.61 ± 0.66‰
Spruce site	Brachycera C	11	-26.59 ± 0.81‰	6.06 ± 1.17‰
Pine site	*Araneus diadematus*	5	-25.26 ± 0.49‰	5.20 ± 1.57‰
Pine site	Pollen *Pinus sylvestris*	18	-26.80 ± 0.16‰	2.52 ± 0.25‰
Pine site	Aphididae	5	-27.73 ± 1.05‰	2.53 ± 0.62‰
Pine site	Hymenoptera	6	-26.82 ± 1.68‰	4.35 ± 1.61‰
Pine site	Nematocera	3	-27.98 ± 0.76‰	6.35 ± 0.71‰
Pine site	Syrphidae	3	-26.22 ± 1.40‰	1.15 ± 1.58‰
Pine site	Brachycera D	4	-27.87 ± 0.61‰	6.21 ± 0.79‰
Pine site	Brachycera E	4	-25.61 ± 0.60‰	3.69 ± 0.91‰
Pine site	Brachycera F	5	-26.14 ± 1.47‰	7.54 ± 0.30‰

Inclusion of the prey groups in the mixing models was based on the literature and their relative abundance. Nyffeler and Benz [18,19] and Nyffeler [20] stated that *A. diadematus* (and many other orb-weavers) most frequently catch very common small dipterans (70-90%) followed by winged aphids (4-16%) with diet principally consisting of only 1-3 insect groups. Due to the fact that an orb-web randomly filters prey out of the aerial plankton [18], very common dipterans should be caught most frequently.

**Figure 2:**
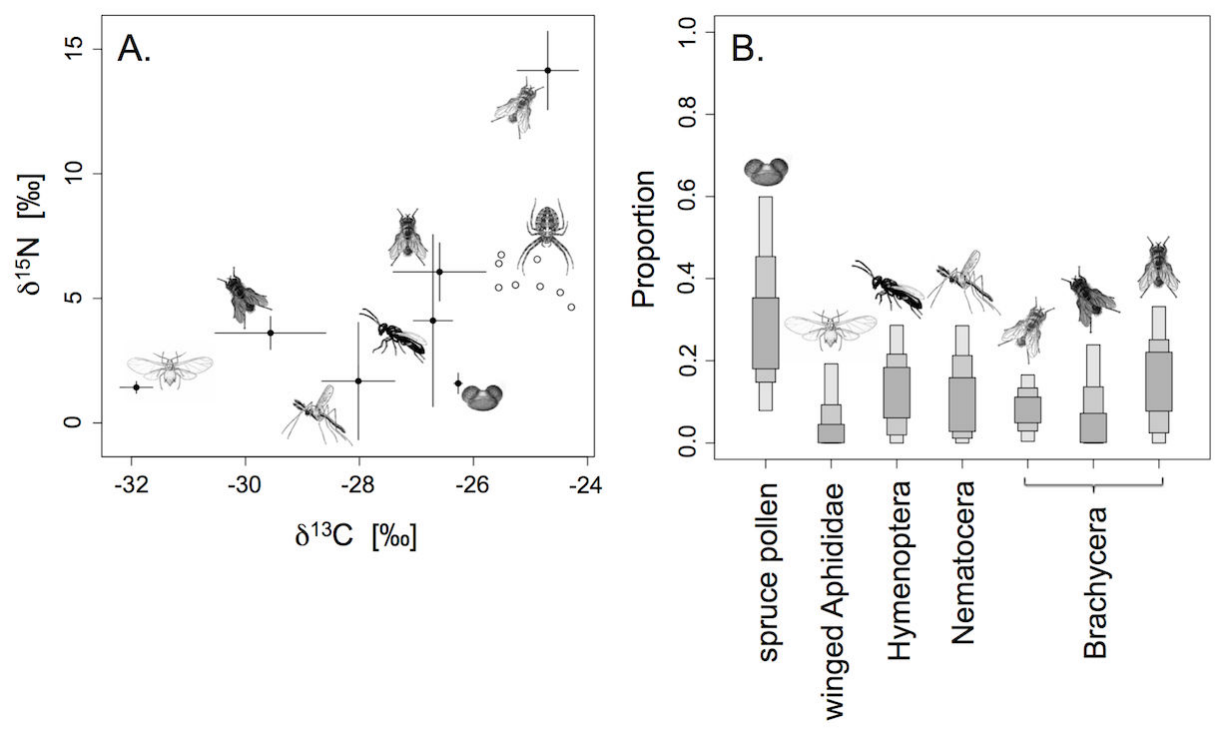
Stable isotope diet analysis of field collected *A. diadematus* and their potential resources at the spruce site. A. δ^15^N and δ^13^C values (mean ± SD) of field collected juvenile *A. diadematus* and their potential resources. The spiderlings are depicted as individuals. B. Composition of the spider’s diet based on Bayesian mixing models. The bars represent 25%, 75% and 95% confidence intervals (CIs) of the single resources the spiders incorporated.

According to our mixing model, aphids were not an important resource for *A. diadematus* at the spruce site (6.36 ± 6.04% of the total diet). These spiders fed mainly on Hymenoptera (13.71 ± 8.41%), Nematocera (13.82 ± 8.56%) and the different Brachycera (about 34%; A: 8.72 ± 4.50%; B: 8.85 ± 7.38%; C: 16.43 ± 9.75%), with spruce pollen being an equally important resource (32.12 ± 13.86%) at this life stage (Figure 2.). At the pine site the spiders fed in part on Hymenoptera (12.49 ± 7.74%) and Aphididae (11.13 ± 7.35%), but mainly on dipterans: Nematocera (8.31 ± 6.33%), and Brachycera (about 54%; Syrphidae: 17.28 ± 8.37%; D: 8.50 ± 6.43%; E: 16.89 ± 8.74%; F: 11.68 ± 7.38%). Again, pollen was an important resource (13.71 ± 7.89%).

## Discussion

The feeding experiment with *A. ceropegia* delivered direct proof that orb-weaving spiders (Araneidae) indeed feed on pollen captured in the sticky spirals of their webs and incorporate this into their body tissue, even when prey is available. The feeding experiment and field samples suggest that about 25% of the spiders’ diet consisted of pollen and the other 75% consisted of flying insects, mainly small dipterans and hymenopterans. The amount of pollen consumption was quite similar in the laboratory and in the field (10-40%), indicating that orb-weaving spiders actively feed on pollen for optimal nourishment, with all essential nutrients delivered by insect prey and resources provided by plants, at least at early life stages. Most pollen grains (so are birch, pine and spruce pollen) are too large to pass through the spider’s pharynx and therefore cannot be swallowed accidentally but have to be actively consumed. Spiders dissolve the outer coating of a pollen grain via extraoral digestion and suck up the dissolved nutrients afterwards [3,5,8, personal observations]. The pollen grains used in this study were of different shapes and sizes (ranging from 25 μm to 75 μm). It is likely that other araneid species feed on different pollen. Therefore pollen, when available, is indeed a very important part of the diet for these juvenile orb-weavers, but not the main food as postulated by Smith & Mommsen [3]. Differences in nutritional value or digestibility can lead to preferences and different rates of pollen uptake in araneids.

The different feeding treatments (pollen+fruit flies vs fruit flies only) did not influence weight gain in juvenile *A. ceropegia* over the month they were fed in the laboratory experiment. This suggests that when insect prey is also available, the supplement through pollen might not be important for spider growth, but it could have an effect on long-term fitness, i.e. reproduction and survival, as indeed pollen has been shown to increase the survival of otherwise starving spiderlings [3]. Therefore, it is likely that pollen availability is crucial for spider survival at the early juvenile stage in spring, when insect prey is scarce in contrast to pollen. The importance of pollen in nutrition may decrease over time, when insect prey becomes more abundant in summer and the flowering season of the most abundant plants is over. However, feeding on pollen as juveniles is sufficient to classify these spiders as omnivores, rather than being pure predators, as they are carnivores and herbivores in an important life stage.

The question remains whether spiders choose their web building location based on pollen availability in the environment. The juvenile *A. diadematus* we observed in this study had built their webs in the branchwood of different wind-pollinated (anemophilous) trees. This could be due to the fact that these are optimal locations for successfully capturing flying insect prey; however it could also be possible that webs are positioned according to pollen availability and spiders select these locations as juveniles. Further studies investigating pollen feeding at different life stages and in different habitats are needed to increase our understanding of the importance of herbivory for spider fitness, behaviour (web-building site choice and web recycling) and their role in ecological communities.

## References

[1] Wise DH (1993) Spiders in ecological webs. Cambridge: Cambridge University Press.

[2] Nyffeler M (1999) Prey Selection of Spiders in the Field. Journal of Arachnology 27: 317-324.

[3] Smith RB, Mommsen TP (1984) Pollen Feeding in an Orb-Weaving Spider. Science 226: 1330-1332. doi:10.1126/science.226.4680.1330. PubMed: 17832631. 17832631

[4] del Fiol F, Solveig T, Riccardo G (2007) Fungal spores and pollen as potential nutritional additives for the cross spider Araneus diadematus Clerck (Araneae, Araneidae). Boletín Micológico 22: 47-50.

[5] Peterson JA, Romero SA, Harwood JD (2010) Pollen interception by linyphiid spiders in a corn agroecosystem: implications for dietary diversification and risk-assessment. Arthropod-Plant Interactions 4: 207-217. doi:10.1007/s11829-010-9106-3.

[6] Sanders D (2013) Herbivory in spiders; Nentwig W Berlin, Heidelberg: Springer Berlin Heidelberg. 385–391 p

[7] Foelix RF (2011) Biology of spiders. Oxford: Oxford University Press.

[8] Ludy C, Lang A (2006) Bt maize pollen exposure and impact on the garden spider, Araneus diadematus. Entomologia Experimentalis et Applicata 118: 145-156. doi:10.1111/j.1570-7458.2006.00375.x.

[9] Roulston TH, Cane JH (2000) Pollen nutritional content and digestibility for animals. Plant Systematics and Evolution 222: 187-209. doi:10.1007/BF00984102.

[10] Post DM (2002) Using Stable Isotopes to Estimate Trophic Position: Models, Methods, and Assumptions. Ecology 83: 703-718. Available online at: doi:10.1890/0012-9658(2002)083[0703:USITET]2.0.CO;2

[11] Ponsard S, Arditi R (2000) What Can Stable Isotopes (δ15N and δ13C) Tell about the Food Web of Soil Macro-Invertebrates? Ecology 81: 852-864. Available online at: doi:10.1890/0012-9658(2000)081[0852:WCSINA]2.0.CO;2

[12] Hood-Nowotny R, Knols BGJ (2007) Stable isotope methods in biological and ecological studies of arthropods. Entomologia Experimentalis et Applicata 124: 3-16. doi:10.1111/j.1570-7458.2007.00572.x.

[13] Oelbermann K, Scheu S (2002) Stable isotope enrichment (δ15N and δ13C) in a generalist predator (Pardosa lugubris, Araneae: Lycosidae): effects of prey quality. Oecologia 130: 337-344. doi:10.1007/s004420100813. 28547039

[14] Jackson AL, Inger R, Parnell AC, Bearhop S (2011) Comparing isotopic niche widths among and within communities: SIBER – Stable Isotope Bayesian Ellipses in R. J Anim Ecol 80: 595-602. doi:10.1111/j.1365-2656.2011.01806.x. PubMed: 21401589. 21401589

[15] R Development Core Team (2011) R: A language and environment for statistical computing. Vienna: R Foundation for Statistical Computing.

[16] Parnell A, Jackson A (2011) siar: stable isotope analysis in R.

[17] Wise DH, Moldenhauer DM, Halaj J (2006) Using Stable Isotopes to Reveal Shifts in Prey Consumption by Generalist Predators. Ecol Appl 16: 865-876. Available online at: doi:10.1890/1051-0761(2006)016[0865:USITRS]2.0.CO;2. PubMed: 16826987 16826987 10.1890/1051-0761(2006)016[0865:usitrs]2.0.co;2

[18] Nyffeler M, Benz G (1981) Freilanduntersuchungen zur Nahrungsökologie der Spinnen: Beobachtungen aus der Region Zürich. Anzeiger für Schädlingskunde, Pflanzenschutz, Umweltschutz 54: 33-39. doi:10.1007/BF01905916.

[19] Nyffeler M, Benz G (1982) Spinnen als Prädatoren von landwirtschaftlich schädlichen Blattläusen. Anzeiger für Schädlingskunde, Pflanzenschutz, Umweltschutz 55: 120-121. doi:10.1007/BF01902580.

[20] Nyffeler M (1983) Eine Notiz zur ökologischen Bedeutung der Radnetzspinnen als Blattlausprädatoren in Gärten. Mitteilungen der Schweizerischen Entomologischen Gesellschaft p. 200. p.

